# Metacognitive Therapy of Early Traumatized Patients With Borderline Personality Disorder: A Phase-II Baseline Controlled Trial

**DOI:** 10.3389/fpsyg.2019.01694

**Published:** 2019-07-30

**Authors:** Hans M. Nordahl, Adrian Wells

**Affiliations:** ^1^Department of Mental Health, Norwegian University of Science and Technology, Trondheim, Norway; ^2^Division of Psychiatry, Nidaros DPS, St. Olav’s University Hospital, Trondheim, Norway; ^3^School of Psychological Sciences, The University of Manchester, Manchester, United Kingdom; ^4^Greater Manchester Mental Health NHS Foundation Trust, Prestwich, United Kingdom

**Keywords:** borderline personality disorder, early childhood abuse, metacognitive therapy, rumination, self-harming behavior

## Abstract

Metacognitive therapy (MCT) is proving to be an effective and brief treatment for anxiety disorders and depression, but there are no investigations of its feasibility and effect on primary personality disorders. We conducted a baseline controlled phase II trial of MCT on a group of patients with Borderline personality disorder all reporting early trauma history with sexual or physical abuse. All had been referred to our study after hospitalization and subsequently treated at the university outpatient clinic at NTNU. Twelve patients referred for severe long-term trauma and emotional instability were offered participation in the program. All gave their consent and were included in the trial. We aimed to examine retention over treatment and follow-up, if the treatment can be delivered in a standardized way across complex and heterogeneous patients and any evidence associated with treatment effects on a range of measures to inform subsequent trials. We measured change in mood, borderline-related symptoms, interpersonal problems, trauma symptoms, suicidal thoughts and self-harming behaviors across pre- post-treatment and by 1- and 2-year follow-up. Treatment appeared feasible with all patients completing the course and 11 out of 12 completing all follow-up assessments. All outcome measures showed a high retention rate and no drop-outs from the treatment. Large improvements over time and treatment gains were maintained at 2 years. There was significant reduction of borderline symptom severity, interpersonal problems and trauma symptoms from pre to 2-year follow-up. The results indicate that MCT may be applied to Borderline personality disorder and that future more definitive trials are warranted.

## Introduction

Patients with Borderline personality disorder (BPD) may be characterized with instability in affect, behavior and self-esteem. They struggle typically with self-destructive forms of impulsivity and typically report a pattern of life-long unstable and dysfunctional relationships, volatile negative affect consisting of anger and depression with self-harming behaviors and suicidal ideation (Oldham, 2006). These problems may occur as acute exacerbations leading to injuries and premature death (Black et al., 2004). Borderline personality disorder is associated with many comorbid disorders, typically traumatic stress, drug abuse, dysphoria, or recurrent depression (Zanarini et al., 2004). This group of patients are in need of targeted interventions to deal with intense dysphoric mood, dysfunctional behaviors, and risk.

The current comprehensive approaches to Borderline personality disorder (BPD) are Dialectical Behavior Therapy (DBT; Linehan, 1993), Schema Therapy (ST; Young et al., 2003) and Transference Focused Psychotherapy (TFP; Kernberg et al., 2002), and more recently Mentalization Based Therapy (MBT; Bateman and Fonagy, 2006). A supplementary or adjunct treatment program called STEPPS is also recently in use (Blum et al., 2008). STEPPS consists of psychoeducation and cognitive behavioral approaches in a package consisting of both individual and group based interventions. Most of these treatments may be categorized as integrative, as they use a broad range of strategies that encompass a wide variety of techniques drawn from different approaches (Lieb et al., 2004). There are indications of beneficial effects of comprehensive psychotherapies as well as non-comprehensive psychotherapeutic interventions for BPD, however, the treatments are often long-term and resource demanding with a high relapse rate (Cristea et al., 2017).

There are several common features shared by the widely used comprehensive therapies. All of them emphasize the therapeutic relationship and validation, structure and directedness, with focus on interpersonal difficulties, management of emotional distress and associated self-harming behaviors (SHB) or suicidal risks (Stoffers et al., 2012). There is therefore no surprise that these treatments have generally equal outcomes at post-treatment and by 12 months follow-up on a variety of borderline-relevant domains (Clarkin et al., 2007; Bateman et al., 2015). Based on the similarity in content and equal effect sizes, there is currently no single treatment of choice for BPD.

Metacognitive therapy (MCT; Wells, 2009) is proving to be an effective treatment for anxiety and depression disorders, with emerging evidence it could be more effective than cognitive behavioral approaches (Nordahl et al., 2018; Normann and Morina, 2018). This raises the possibility that it might also be useful in borderline personality disorder patients, who show long term difficulties regulating anxiety and mood.

So far Metacognitive therapy has not been systematically applied with primary personality disorder, but it targets core transdiagnostic processes in psychopathology that should be evaluated in a treatment trial. We therefore adapted the principles of the self-regulatory executive function model (Wells and Matthews, 1996) and metacognitive therapy for anxiety and depression (Wells, 2009) to develop a treatment protocol of BPD.

The resulting protocol offers a brief targeted treatment for patients with BPD or borderline personality spectrum disorder. It consists of several components. First, the preparation and formulation of contracts, shaping of the patient’s expectation of therapy and planning of collaboration and the main tasks. Second, a metacognitive-focused modification of self-defeating beliefs and strategies, third targeting executive functions, including de-centered responding to negative thoughts; fourth, involvement of the community psychiatry service in general psychiatric management by the end of therapy and in the following time after the therapy. In addition, where applicable the family and caregivers are involved in order to facilitate family life and support the patient so they will be encouraged and committed to attend the program. In this study we planned to deliver sessions for a maximum of 12 months and the patients were asked to sign a contract that that had been informed and consented to this requirement before entering into the treatment. All patients were offered continuation in general health care management at community health care centers after the 12 months treatment phase was completed.

The goal of the current study was to explore the feasibility, tolerability and preliminary evidence of treatment associated effects of the protocol, also called a phase-II trial. In the current phase-II baseline-controlled trial each patient acted as their own control and we conducted an exploratory assessment of outcomes, which were measured prior to therapy, at post-treatment, at 1- and 2-years after treatment. In this study we were able to examine the feasibility, symptom change at various stages and the long-term effects. The drop-out rate and level of attendance was used as the primary indication of feasibility and tolerability.

Patients with borderline personality, especially those requiring hospitalization are often more complex with diverse and multiple pathologies. As this was our target group we selected a range of measures of outcome, we wanted to see if changes could be observed across more specific but also general measures especially those that assess risk, trauma symptoms, and quality of life.

## Materials and Methods

### Design

Patients with early experiences of sexual or violent traumas and BPD were recruited and treated in this open trial. They were all referred to our outpatient clinic for treatment after being hospitalized. All patients received comprehensive treatment according to the protocol and each acted as their own control. To optimize the design of the study we applied a systematic replication design with measures at baseline (4 times over 6 weeks), in which each patient acted as their own control at pre-treatment. We assessed again at post-treatment and at follow up by 1 and 2 years. Baseline measures were ratings of depression and anxiety symptoms (BDI-II and BAI). We measured the change in borderline-related symptoms (structured interview of the DSM-IV criteria), interpersonal problems (IIP-64), symptom severity (SCL-90-R, BDI, and BAI), post-traumatic symptom criteria (PDS), the metacognitive beliefs and cognitions (ERIS) and quality of life (WHO-5) once at pre-treatment and at 1- and 2-year follow-up.

### Subjects

Twelve inpatients from local psychiatric hospitals were subsequently referred after hospitalization and treated at the university outpatient clinic 2007–2012. Of the 12 patients 10 were females (83%) and 2 were males. Mean age was 32.08 (range 19–51). In the group 50% were single, 25% were married or co-habitant and 25% were divorced/separated. Two-thirds of the sample were unemployed or students/trainees of work placement. The rest were in part-time jobs or on disability pension. The mean years of previous treatment including both outpatient treatment and being hospitalized were 7.2 years, and 83% (10/12) were currently treated with psychopharmacology. The distribution of medication was: Neuroleptics (33%), antidepressants (58%), antiepileptic (25%), and benzodiazepines (25%). The patients taking drugs had been stabilized on medication after discharge from the hospital, and they agreed to carry on with the same dosage during the treatment, unless the GP or psychiatrist suggested otherwise.

To be included they had to read and sign a form declaring the rules and time frame of the program. The inclusion criteria were; (1) Borderline personality disorder as primary disorder, (2) age 18 or older, (3) they should consent to the time frame of the treatment program of maximum 12 months and be committed to follow the outpatient treatment on a regular basis. The only exclusion criteria were having somatic illness that needed continuous medical attention, having an active psychosis or substance addiction (alcohol or drugs).

All referred patients accepted these terms after a thorough briefing and signed the contract of participation in the program. None of the referred patients were excluded. Self-harming behaviors and suicidality was assessed by an independent assessor at all time-points of evaluation. This was also monitored through self-report questionnaires during the treatment. This monitoring was a safeguard and an outcome to decide if the patient at any time was self-harming or planning suicide or changed their inclination to act on those thoughts.

The mean number of additional ADIS-IV diagnoses found in this sample was above 4 (*M* = 4.7). Five patients fulfilled criteria for 6 disorders, and 3 patients fulfilled 5 disorders. Two patients had only 2 disorders, and 2 patients had 3 and 4 disorders, respectively.

The most frequent additional disorders were 12 patients (100%) with anxiety disorder (Social anxiety disorder or Panic disorder/Generalized anxiety disorder), 10 patients (83%) fulfilled the criteria of chronic PTSD, 8 had recurrent depression (67%), and 4 had substance abuse (25%). See Table 1.

**TABLE 1:** Demographic characteristics of the sample (*N* = 12).

**Index**	**Category**	**Mean**	**SD**	**N (%)**
Age		32,08	11,73	12 (100)
Sex	Female			10 (83)
	Male			2 (17)
Status: familial	Single			6 (50)
	Married			3 (25)
	Divorced			3 (25)
Status: work	Unemployed			3 (25)
	Part time job			2 (16)
	Full time job			0 (0)
	Student/trainee			5 (41)
	Disability pension			2 (16)
DSM-V diagnosis	Social phobia			3 (25)
	GAD			2 (16)
	Panic disorder			3 (25)
	Specific phobias			4 (33)
	MDD recurrent			8 (67)
	Eating Disorder NOS			4 (33)
	Substance abuse disorder			4 (33)
	PTSD, chronic			10 (83)
	Dissociation			3 (25)
	Somatoform disorder			4 (33)
	Cluster A PD			2 (16)
	Cluster B PD^*^			4 (33)
	Cluster C PD			6 (50)

^*^Other than BPD. GAD, Generalized Anxiety Disorder; MDD, Major Depressive Disorder; PD, Personality Disorder.

### Primary and Secondary Measures

The primary outcomes were the drop-out and attendance rates for patients across treatment. The secondary outcomes were specific (borderline-related symptom criteria; SCID-II criteria SCID-II; First et al., 1997/2004) and general symptom severity, impact on processes of worry, rumination and metacognitions, quality of life and risk.

### Measures

*The inventory of interpersonal problems* (IIP-64), the 64-item version was used to measure various problem areas in interpersonal dysfunction with a five-point Likert-type scale. High scores for the total scale and for its 8 subscales indicate an increased level of interpersonal problems and distress. The internal consistency coefficient (Cronbach’s Alpha) and test-retest reliability for the original inventory were 0.93 and 0.78, respectively (Horowitz et al., 1988).

*The post-traumatic stress diagnostic scale* (PDS; Foa, 1996; Foa et al., 1997) was developed and validated to provide a brief but reliable self-report measure of post-traumatic stress disorder (PTSD) for use in both clinical and research settings. The scale is intended to screen for the presence of PTSD in patients who have identified themselves as victims of a traumatic event or to assess symptom severity and functioning in patients already identified as suffering from PTSD. The test is self-administered and requires a reading age of >12 years.

*Emotional and relationship instability scale* (ERIS; Nordahl and Wells, 2009b), is a rating scale developed to measure borderline-relevant symptoms and beliefs, in particular the patients psychological distress related to abandonment and rejection. Also ERIS measures maladaptive coping behaviors (CAS) and metacognitive beliefs associated with maladaptive coping. It is one of the few self-report measures designed for patients with borderline personality and it uses a Likert scale from 0 to 8 (0 = None of the time, 8 = Every time). In a study of 133 patients with borderline spectrum personality, we found that ERIS possesses a reliability (internal consistency) of ICC = 0.91, with a three-factor latent structure that explained 48% of the scale variance. The psychometric properties of the ERIS were satisfactory (Bruset Ludviksen, 2014).

*WHO-5 well-being index*, is a brief questionnaire, which consists of 5 questions tapping the subjective well-being of the respondents. The scale is derived from other rating scales, and each of the 5 items is scored from 5 (all of the time) to 0 (none of the time), so the range is from 0 to 25, indicating maximal well-being. The WHO-5 is widely used and has adequate predictive validity both as a screening tool and an outcome measure in clinical trials (Topp et al., 2015).

### Procedure

All patients were assessed with ADIS-IV (Di Nardo et al., 1994) and SCID-II (First et al., 1997/2004) by independent clinicians at the outpatient clinic. As well as diagnosis, the severity of their borderline disorder, the inclusion and exclusion criteria were assessed for each patient by independent assessors at the university clinic. The patients were given both oral and written information about the study and implications of participating. All patients gave written consent in order to participate in the study. They then completed four baseline data assessments of anxiety and depression before the treatment was provided, and at the pre-treatment stage the full set of measures were administered to each of the subjects. This set of measures was administered again at post-treatment, and by 1- and 2-year follow-up.

### Treatment

The first phase in the protocol was to negotiate a contract and shape the patient’s expectation about his/her and the therapist’s role in the program. In addition, there was some planning of the collaboration and availability of the therapist and early involvement of the community service. The second and the third phase focused on self-defeating beliefs and the self-regulatory executive functions (Wells and Matthews, 1994) of the patient:

The following steps were implemented:

(1) Formulation and socialization.
(2) Eliminate the impact of self-defeating factors.
(3) Increase cognitive flexibility and attentional control.
(4) Reducing maladaptive coping strategies (worry, rumination, and threat monitoring)
(5) Modify negative and positive metacognitive beliefs
(6) Alternative strategies and new personal goals.

The formulation (1) was shared with the patient, and the therapist socialized to the treatment. The socialization is necessary to help the patient to understand their distress in a metacognitive framework. The aim of the formulation and socialization is to develop a common understanding of the problems and the basis for the interventions. The self-defeating factors (2) are conceptualized as a set of unhelpful metacognitive beliefs about control (“I cannot control my mind;” “I cannot stop ruminating”), or change (“my mind is broken” or “the problem is in my genes”). They also include beliefs about the value of negative self-referential thinking (“I need to put myself down in order to feel safe” or “only by punishing myself I can feel okay”). These self-defeating metacognitions can work against any treatment engagement and recovery.

Self-harming behaviors and suicidal threats are also self-defeating factors and should be addressed specifically in these sessions. We helped the patient be explicit about them, even if it is subjectively shameful and normally avoided. In this early work the therapist helped the patient to develop a sense of responsibility for his/her actions; as this is something the patient chooses to do to handle distress and negative thoughts (i.e., labeled as an unhelpful coping strategy).

An advantage/disadvantage analysis was run in collaboration with the patient, and the therapist explored if there might be more beneficial ways to deal with life that might involve learning how to reduce worry and self-punishment. The therapist and patient made an agreement to stop acting on self-harming beliefs and behaviors and to test alternatives. A new plan and alternative strategies was developed, monitored and followed-up in sessions until the patient had modified the beliefs and behaviors that interfere with the goals of treatment.

Many borderline patients worry about rejection and abandonment in social relationships, and they ruminate about past events (e.g., being ignored) and losses (3) Rumination is a strategy, which involves dwelling on past failures, abandonments, and criticisms (Nolen-Hoeksema, 1991). Rumination has different forms, and the most prominent for borderline patients is depressive and angry rumination. Angry rumination consists of repetitive thoughts about the unfairness of life, where the patient experiences that they are scapegoated, unjustly blamed or not “understood” by others. This creates frustration and anger and feelings of being criticized, attacked or alone. Depressive rumination is about past failures, abandonments and losses and creates a self-critical, self-blaming, and depressed mood. Worry, in contrast consists of anticipating rejection, abandonment or loss of credibility, and this leads to anxiety and fear and the tendency to avoid or sacrifice relationships. Rumination and worry are seen as central maintenance processes in the metacognitive model and thus, an important intervention in MCT is to help the patients to reduce the level of worry and ruminations, as these contribute to anxiety, dysphoria or depressive mood.

Cognitive flexibility (4) is the ability to selectively control the focus of attention and to respond to worry and rumination by moving attention away from inner or external threatening stimuli. In order to be cognitively flexible, the patient must work on postponing responses, and develop greater awareness of choice in whether or not to respond to internal (thoughts, feelings) or external events (e.g., being ignored). The treatment protocol applies the Attention training technique (ATT; Wells, 2009) as this is designed to change the metacognitions that regulate thinking and facilitate emotional processing by interrupting excessive self-focused attention and brooding. Detached Mindfulness (DM; Wells, 2005), is also used as this technique helps the patient to explore and discover their executive control of thinking (5). The essential idea in DM is to leave alone any cognition even when triggered by some distressing thoughts or feelings. In this way the patient can refrain from perseverative thinking such as rumination, worry or threat monitoring, and can be encouraged to choose to postpone these processes.

Improving the patient’s functioning in work and relationships are the primary targets in setting new personal goals (6). The therapist worked with the patient in developing concrete goals in these areas. Even though the acute-phase treatment program finished after 12 months, we took a 2-year perspective of working toward a better life context within these areas. The therapist discussed the following with patients: “Where do you want to be in your life in 2 years from now in terms of: education? substance abuse? relationship to parents? dealing with your mood? contact with friends?” and other relevant domains. The advantages of working toward these goals were explored and a step by step concrete plan was formulated. The implementation of these goals was a recurrent issue during the program, and both the patient and the therapists monitored the progress toward these concrete and within reach goals.

The last phase of the protocol comprised transferring the patient to general community psychiatric management. This is a team consisting of a family therapist, or psychiatric nurse and general practitioners responsible for the support and follow-up of the patients. The main therapists continued to support the team in a role as a clinical supervisor. The main task of the community management team was to follow up the goals and adaptive strategies from treatment and to help and support the patient in the job or school situation. The general psychiatric service continues to follow-up the patient under monthly supervision of the therapist. The patients do not attend these meetings, but are informed by the psychiatric nurse. Normally the general psychiatric management follows the patient from 1 to 2 years after treatment, but this is based on individual needs.

### Therapists and Treatment Integrity

The treatment was conducted by two experienced therapists each of whom have 20 years of clinical practice and training in metacognitive therapy. Overall supervision in MCT was provided by AW and site supervision by HMN. Treatment followed a draft protocol by the authors and adherence and the level of competency was monitored by the Metacognitive therapy competency scale (MCT-CS; Nordahl and Wells, 2009a).

### Statistical Analysis

Primary outcome and secondary outcome measures were subjected to repeated measures ANOVA, with time (pre-treatment, post-treatment, 1 and 2-year follow-up) as the repeated measures factor.

We used Hedge’s *g* to estimate the effect sizes between pre- and post-treatment and between pre-treatment to 2-year follow-up (Hedge, 1981). Hedge’s ***g*** is attained by subtracting the post-treatment means from the post-waitlist means and dividing this by the pooled standard deviation and correcting for the sample size (*N* < 20). Missing data was imputed by using unit imputation substituting the missing value by the mean of the observed values for that variable.

## Results

All patients (*N* = 12) attended 12 months of therapy consisting of up to 40 sessions (range 20–45) and we had a 100% completion rate. There were no drop-outs during the acute treatment phase. No suicidal attempts were reported, although suicidal thoughts and self-injury occurred during treatment but showed a decline in severity (see Figure 2). All patients completed the 1 year follow-up measures, but 11 of 12 filled in the measures at 2-year follow-up. One patient was lost to 2-year follow-up and did not fill in the questionnaires as we were unable to get in contact with her.

### Feasibility and Retention

The patients reported in interview that they experienced the treatment to be helpful and meaningful to them reporting that the rational for the treatment made sense. The mean number of treatment sessions were *M* = 26.6 sessions (*SD* = 6.15) and the time frame was between 9 and 14 months (*M* = 11.5). There were no dropouts from pre- to post-treatment and there was a high retention rate where all attended between 70 and 90% of the sessions offered. The transition to community psychiatry management seemed to work well in most patients, and 8 of the 12 patients made use of this service after treatment and by 2-years follow-up 4 patients were still in contact with the community management service on a regular basis.

### Baseline Change

**FIGURE 1:**
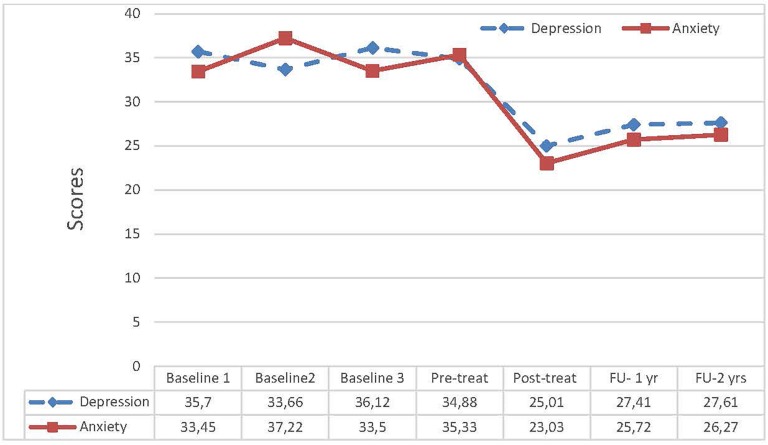
Levels depression and anxiety from baseline to 2-year follow-up.

A repeated measures ANOVA was used to examine any changes in symptom level during baseline. Neither anxiety nor depression changes were significant during the baseline period [BAI; *F*(3,15) = 1.140, *p* = 0.354; BDI-II; *F*(3,15) = 1.584, *p* = 0.357], indicating that there were no major or systematic changes in anxiety and mood occurring during the pre-treatment period (see Figure 1).

### Outcome of Borderline Related Symptoms

One-way repeated measures ANOVAs were run for each of the outcome variables across pre-treatment, post-treatment and 1- and 2-year follow-up (*N* = 12). Mauchley’s test of spherity was not significant and thus not violated, so adjustments were not needed. However, due to multiple *post hoc* analyses we used Bonferroni correction in the analysis of the main measures. The results indicated a significant time effect for borderline-related symptom criteria, Wilks’ Lambda = 0.085, *F*(3,30) = 27.991, *p* < 0.001, $\eta_{p}^{2}$ = 0.737, showing a significant reduction. The pairwise comparisons between pre and post-treatment showed a significant reduction (*p* = 0.001), and from pre to 1-year follow-up (*p* < 0.001) and from pre to 2-year follow-up (*p* = 0.003).

For the changes in depression across time the effects were as follows: Wilks’ Lambda = 0.24, *F*(3,27) = 15.676, *p* < 0.001, $\eta_{p}^{2}$ = 0.635. The pairwise comparisons here were from pre- to post-treatment (*p* = 0.007), from pre-treatment to 1-year follow-up (*p* = 0.013), from pre-treatment to 2-year follow-up (*p* = 0.009). For anxiety the results showed large reductions, Wilks’ Lambda = 0.123, *F*(3,27) = 28.376, *p* < 0.001, $\eta_{p}^{2}$ = 0.759. The pairwise comparisons were; pre- to post-treatment (*p* < 0.001), pre-treatment to 1-year follow-up (*p* < 0.001), and pre-treatment to 2-year follow-up (*p* = 0.004).

Significant reductions across time were also observed for other symptom domains such as, interpersonal dysfunction, Wilks’ Lambda = 0.104, *F*(3,27) = 30.247, *p* < 0.001, $\eta_{p}^{2}$ = 0.771.

The pairwise comparisons were; pre-treatment to post-treatment (*p* < 0.001), pre-treatment to 1-year follow-up (*p* = 0.001), and pre-treatment to 2-year follow-up (*p* < 0.001).

Post-traumatic symptoms, Wilks’ Lambda = 0.203, *F*(3,30) = 13.643, (*p* = 0.004), $\eta_{p}^{2}$ = 0.577.

The pairwise comparisons were; pre-treatment to post-treatment (*p* = 0.001), pre-treatment to 1-year follow-up (*p* = 0.003), and pre-treatment to 2-year follow-up (*p* = 0.018).

Level of worry/rumination, Wilks’ Lambda = 0.046, *F*(3,30) = 54.187, *p* < 0.001, $\eta_{p}^{2}$ = 0.844.

The pairwise comparisons were; pre-treatment to post-treatment (*p* < 0.001), pre-treatment to 1-year follow-up (*p* < 0.001), and pre-treatment to 2-year follow-up (*p* < 0.001).

Quality of life (WHO-5), Wilks Lambda = 0.196, *F*(3,30) = 11.022, (*p* = 0.001), $\eta_{p}^{2}$ = 0.542. The pairwise comparisons were; pre-treatment to post-treatment (*p* = 0.016), pre-treatment to 1-year follow-up (*p* = 0.003), and pre-treatment to 2-year follow-up (*p* = 0.032). The means and standard deviation for the outcome measures across time are presented in Table 2.

**TABLE 2:** Means and standard deviation for the sample (*N* = 12) and changes from pre-treatment to 2-year follow-up across time!

**Index**	**Pre-treatment**	**Post-treatment**	**Follow-up 1**	Follow-up 2^*^	***p***	**Hegde’s g (T_1_-T_2_)**	Hegde’s g^*^ (T_1_-T_4_)
BPD severity (M) (SD)	6.25	3.75	3.58	4.18	<0.001	2.89	1.93
	1.14	1.35	0.90	1.32			
BDI-II (M) (SD)	35.83	25.01	27.41	27.60	<0.001	1.44	1.26
	5.87	8.94	8.34	7.61			
BAI (M) (SD)	35.33	23.83	25.72	26.27	<0.001	2.31	1.42
	5.34	4.95	4.40	5.60			
IIP-64 (M) (SD)	134.33	111.41	114.58	109.30	<0.001	1.01	1.38
	21.57	19.90	17.61	12.50			
PDS (M) (SD)	28.16	21.41	20.33	20.18	0.004	0.72	1.09
	8.37	8.41	7.16	5.30			
ERIS (M) (SD)	6.50	2.75	2.91	2.63	<0.001	3.26	2.96
	1.38	1.21	1.08	1.12			
QoL (M) (SD)	5.91	8.75	9.23	8.54	0.001	1.45	1.13
	2.67	2.73	2.66	1.69			

^*^N = 11. Hedge’s g pre-post (*T*_1_-*T*_2_); Hedge’g Pre-2 years FU (*T*_1_-*T*_4_). BPD, Borderline personality disorder; BDI, Beck Depression Inventory; BAI, Beck Anxiety Inventory; IIP-64, Inventory of Interpersonal Problems; PDS, Post-Traumatic Diagnostic Scale; ERIS, Emotional and Relationship Instability Scale; QoL, Quality of Life.

As the sample size was <20, we used corrected effect size (g) using Hedge’s formula (Hedge, 1981). Table 2 shows that the effect sizes were large, and comparable to other comprehensive treatments for patients with Borderline personality disorder (Giesen-Bloo et al., 2006; Clarkin et al., 2007). For both of the outcome measures the effects sizes (g) were between 1.0 and 1.8, whereas the PTSD symptoms showed moderate change from pre-post (*g* = 0.724) and increased from pre- to 2-year follow-up (*g* = 1.09). The levels of self-reported worry/rumination about abandonment/rejection had a significant drop and indicates a major change in the thinking styles in all patients. Also, the QoL well-being index showed that patients overall were more satisfied by the end of treatment (*g* = 1.455) and at follow-up (1.136).

### Levels of Suicidal Thoughts and Self-Harm

**FIGURE 2:**
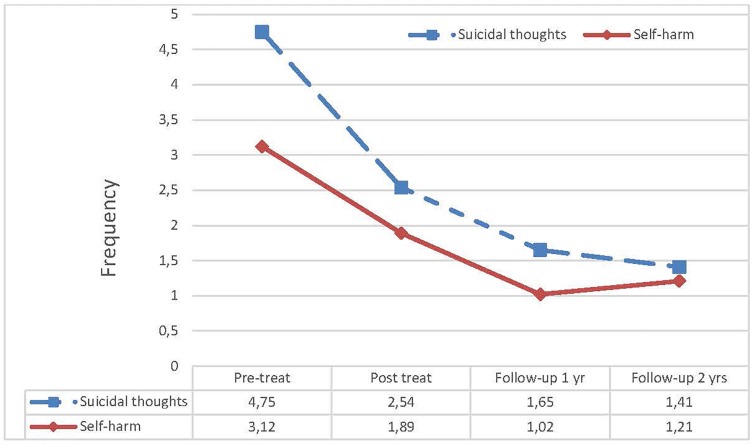
Self-reported self-harming behaviors and suicidal thoughts from pre-treatment level to 2-year follow-up.

The severity of suicidal thoughts/impulses and self-harming behaviors (SHB) was evaluated during the course of treatment by the therapists. The level of reported suicidal thoughts significantly decreased and this improvement held up at 2-year follow-up, Wilks’ Lambda = 0.134, *F*(3,30) = 20.212, *p* < 0.001, $\eta_{p}^{2}$ = 0.669. The pairwise comparisons were; from pre-treatment to post-treatment there was no significant reduction (*p* = 0.115), but from pre-treatment to 1-year follow-up the reduction was significant (*p* = 0.001), and from pre-treatment to 2-year follow-up (*p* = 0.003). The level of self-harming behaviors was also decreasing significantly across time, Wilks’ Lambda = 0.380, *F*(3,30) = 8.625, *p* = 0.007, $\eta_{p}^{2}$ = 0.463. However, the pairwise comparisons showed no significant reduction from pre-treatment to post-treatment (*p* = 0.173), but significant reductions from pre-treatment to 1-year follow-up (*p* < 0.040), and from pre-treatment to 2-year follow-up (*p* = 0.076). See Figure 2.

### Psychopharmacology

The patients taking drugs (*n* = 10) were stabilized on medication after discharge from the hospital, but the users of antidepressant medication and benzodiazepines were tapering their drugs toward the end of the 12 months’ treatment program (*n* = 7).

## Discussion

Feasibility trials are an important first step before applying an intervention to new patient groups as they provide information about tolerability and acceptability of new treatments and indicate whether it should be used or further tested against existing treatment. The results of the current study suggest that this protocol has some important qualities. No patients dropped out in the pre- and post-phase, and only 1 patient was missing at 2-year follow-up. The session attendance rate varied between 70 and 90%, which is highly satisfactory compared to other relevant studies (Landes et al., 2016). Overall most patients were significantly less symptomatic after treatment and upheld the gains during the 1 to 2-year follow-up. The outcome shows large effects sizes and most patients had clinical or subclinical levels of symptoms and functioning at post-treatment. In addition, the interpersonal problems and trauma symptoms and overall well-being showed significant changes, including areas that were not targeted in the intervention. This indicates that metacognitive therapy may be feasible and useful for patients with BPD and early trauma. The protocol applies a coherent and understandable theoretical rational for the treatment, which may be crucial for outcome and for lower attrition or retention (Verheul and Herbrink, 2007).

The preliminary results of our study compare well with other comprehensive treatments in terms of pre- and post-treatment effects and duration (Linehan et al., 2006; Bateman and Fonagy, 2009; Nadort et al., 2009; Doering et al., 2010).

The main target in Transference-focused psychotherapy is the patients’ interpersonal dynamics which is manifested in the transference (misattribution of emotional reactions). Doering et al. (2010) conducted an 18 months’ study of Transference-focused psychotherapy for BPD and used numbers of drop-outs and suicide attempts as the main measures. In this study they found that the Transference-focused treatment was significantly better than treatment conducted by experienced community psychotherapists, but on measures of anxiety and depression levels there were no differences. Typically, two sessions per week are delivered. In the study the dropout rates were high (53%) and the assessments of follow-up had a high proportion missing (38%), which lowers reliability of the findings.

In Schema focused therapy the main target is the healing of early maladaptive schemas and modes (schema clusters). Nadort et al. (2009) conducted Schema Focused Therapy (SFT) in a group of patients with BPD in regular mental health care with an addition of a therapist telephone availability (TTA) all day. They treated the patient over the time span of 18 months, and had a recovery rate of 40%, and effect sizes of 1.5. The treatment effects and drop-out were 22% but no effect of the TTA component was found. These results are comparable to our own study in terms of being brief and well controlled with high effect sizes. However, Schema therapy is designed for 18–36 months of treatment, and involves both individual and group sessions. There are no follow-up studies beyond 12 months, thus the long-term outcome is not known.

In DBT a main emphasis is on the patients’ skills acquisition and behavioral shaping. This is conducted in a context of the dialectic of validation and problem solving. DBT integrates many techniques and is designed to be adapted to a variety of treatment settings and patients. Linehan et al. (2006) compared 12 months DBT with treatment as usual conducted by specialist therapists in BPD and suicidal behaviors, and reported significantly better outcomes of DBT compared to TAU on borderline related symptoms and behaviors. The attrition rate was 25% and 10% lost to 1-year follow-up in the DBT condition. The treatment of DBT is more comprehensive and includes weekly individual therapy with group skills training session, out-of-session paging, and consultation team for the therapist. Thus, DBT has the most intensive and structured scheme of treatment of all the comprehensive therapies.

Mentalization based therapy (MBT) is rooted in attachment theory, theory of mind and psychodynamic principles. The main target is to increase the patient’s capacity to mentalize thoughts and emotions under stress, in order to stabilize cognition in settings of social interactions and emotional distress. It is proposed that the problems of mentalization in patients with BPD may be linked to dysfunctional early attachment (Bateman and Fonagy, 2006). In a study of patients with BPD in an outpatient setting it was reported that an 18 months’ treatment program combining individual and group sessions showed a large improvement in self-injurious behavior, suicidal behavior and hospitalization in the MBT group, and significantly better than in the clinical management comparison, which had an emphasis on social problem-solving (Bateman and Fonagy, 2009). The results were good for borderline-related behaviors, reduction of symptomatic distress, reduced use of medications and improved social functions. Approximately 75% completed the trial, but the data on retention rate (attending sessions) was not available (Bateman and Fonagy, 2009).

The main target in MCT is to improve self-regulatory executive functioning and reduce self-defeating processes. This intervention is different from other approaches used in the treatment of BPD. To work more systematically and directly on the attentional processes and executive functions, but also reducing the level of perseverative thinking, such as angry rumination, is unique to the MCT approach. Furthermore, targeting the cognitive attentional syndrome (CAS) and modifying the self-defeating metacognitive beliefs is at the core of MCT in order to achieve more adaptive self-regulation and cognitive flexibility.

The results in the current trial suggest that MCT was associated with good clinical response from pre- to post-treatment on borderline-related symptoms, mood and interpersonal problems. Also we observed an effect on trauma symptoms, which were not directly targeted in the treatment. The gains seem to be maintained at 2-year follow-up. This relatively brief intervention seems to be feasible for outpatient treatment, and appears to compare favorably with comprehensive treatments of longer duration. For a more comprehensive overview of the outpatient treatments conducted (see Supplementary Table S1).

There are some important limitations in the current study. First, the sample size is small, therefore any direct comparison of the results with larger comprehensive studies must be interpreted with caution. Low N trials have a higher risk of imprecise estimates and inflated effect sizes; the results may not therefore be reliable. Second, even though the participants were well monitored during the trial, by 2-year follow-up, one patient was not possible to locate. Thus, the data on that particular patient is missing and was not included in the analysis at 2-year follow-up. Third, there was no standardized format of care after treatment, as this was adapted to the individual client within the general community setting. The resources put into community care management could be different from one site to another, as this was due to local resources and availability of health care workers. Thus, we cannot estimate the degree of influence on the results this variability might have had at 1 and 2-year follow-up. Fourth, the competency level of the therapists was likely increasing during the trial, as it does in most trials, and this indicates that the adherence and the competency level in the treatment protocol was probably not consistent across the course of the study.

## Conclusion

The MCT protocol evaluated here seems to be feasible and well tolerated by patients with BPD and early trauma. It was associated with significant improvement in a 9–14 months’ program and the symptom reductions were maintained over 2 years after treatment. The treatment effects across various domains indicate a *trans*-symptomatic improvement, which should be explored further. A metacognitive approach in combination with an adapted community service seems very promising and further testing allowing for variability in the length of the program are warranted using larger samples and comparative randomized controlled trial methodology.

## Data Availability

The datasets generated for this study are available on request to the corresponding author.

## Ethics Statement

Ethical approval from the Regional Committees for Medical Research Ethics in Central Norway (REK No. 2011/2528) was obtained for this study.

## Author Contributions

HN initiated and conducted the trial, did most of the treatments, or supervised the co-therapist, carried out the data analysis, and wrote the main draft of the manuscript. AW acted as a supervisor, advised on design and analysis, and contributed to writing the manuscript. Both authors approved the final version of the manuscript.

## Conflict of Interest Statement

HN and AW have received fees for teaching in MCT and CBT and royalties for books within this subject area.
