# Supplementary material for: Metacognitive Therapy of Early Traumatized Patients With Borderline Personality Disorder: A Phase-II Baseline Controlled Trial
Source: Front Psychol. 2019 Jul 30;10:1694. doi: 10.3389/fpsyg.2019.01694 (PMC6682682; doi:10.3389/fpsyg.2019.01694)
Supplement: Supplementary file 1 [file Table_1.pdf]

Table S1. An overview of outpatient treatments of borderline personality disorder since 2005: Duration, attrition, effect size and recovery rate

Authors	Treatments	N[a]	Duration of treatment (months)	Attrition[b]	Effect size [c] measure	ES Pre-post	Recovery rate BPD	Duration of Follow-up (months)	ES Pre-follow-up
Nordahl et al., 2005	ST	6	22	16%	YSQ, IIP, SCL-90, BDI	1.9	50%	12	1.8
Giesen-Bloo et al 2006	ST<br>TFP	44<br>42	36	27%<br>51%	BPDSI (Interview; DSM-IV BPD psychopathology)	2.96[d] *<br>1.85[d] *	46%<br>24%	Na.	Na.
Nadort et al 2009	ST w/P<br>SFT w/out P	32<br>30	18	22%<br>20%	BPDSI (Interview; DSM-IV BPD psychopathology)	1.42 *<br>1.82 *	42%<br>43%	Na.	Na.
Farrell et al 2009	ST & TAU<br>TAU	16<br>12	8	0%<br>25%	DIB-R (Interview; DSM-IV BPD psychopathology)	2.31<br>0.47	94%<br>16%	6	2.37<br>0.37
Davidson et al 2006	CBT<br>TAU	54<br>52	12	3,7%<br>7,7%	YSQ (Self-report; maladaptive schemas)	0.84<br>0.42	Na.	12	0.79<br>0.37
Cottraux et al 2009	CBT<br>RST	33<br>32	12	39%<br>44%	YSQ (Self-report; maladaptive schemas)	1.17<br>1.10	Na.	12	1.99[g]<br>0.55[g]
Linehan et al 2006	DBT<br>ECP	52<br>49	12	19%<br>43%	Suicide ideation (Self-report; suicide behaviour question.)	0.97<br>1.13	Na.	12	1.36<br>1.16
McMain et al 2009	DBT<br>GPM	90<br>90	12	39%<br>38%	ZAN-BPD (Interview; DSM-IV BPD psychopathology)	1.23<br>1.09	Na.	Na.	Na.
Clarkin et al 2007	TFP<br>DBT<br>SUPT	30<br>30<br>30	12	23%<br>43%<br>27%	Suicidality (Self-report; Overt aggression scale modified)	0.70[e]*<br>0.72[e]*<br>0.37[e]*	Na.	Na.	Na.
Doering et al 2010	TFP<br>ECP	45<br>39	12	29%<br>56%	DSM-IV BPD Criteria (Interview; Scid-II)	1.8 *<br>1.4 *	42%<br>15%	Na.	Na.
Bateman et al 2009	MBT<br>SCM	71<br>63	18	27%<br>25%	Hospital admission due to Suicidality and self-injury	1.29<br>0.77	Na.	Na.	Na.
Gregory et al 2008/2010	DPP<br>TAU	15<br>15	12	33%<br>40%	BEST (Self-report; severity of DSM-IV based symptoms)	1.43*<br>0.73*	Na.	18	1.51<br>0.69
Weinberg et al 2006	MACT & TAU<br>TAU	15<br>15	2	0%<br>0%	Frequency of deliberate self-harm	0.81<br>0.58	Na.	6	0.67<br>0.13
Blum et al 2008	STEPPS & TAU<br>TAU	65<br>59	5	31%<br>14%	ZAN-BPD (Interview; DSM-IV BPD psychopathology)	1.21[f]<br>0.53[f]	Na.	12	0.40 [f, h]<br>0.36 [f, h]
Bos et al 2010	STEPPS<br>TAU	42<br>37	4 ½	21%<br>11%	BDP-40 (self-report; DSM-IV BPD psychopathology)	1.06<br>0.19	Na.	6	1.18<br>0.35
Priebe et al., 2012	DBT<br>TAU	40<br>40	12<br>12	52%<br>15%	DSH (Deliberate self-harm)	0.49<br>0.06	Na.	6	Na.
Bohus et al., 2013	DBT<br>TAU	43<br>39	3<br>3	5%<br>5%	CAPS (Trauma symptoms) PDS (PTSD symptoms)	1.50<br>0.10	Na.	6	1.73<br>0.16
Linehan et al., 2015	DBT<br>DBT-S<br>DBT-I	33<br>33<br>33	12<br>12<br>12	24%<br>39%<br>48%	Suicide attempts and NNSI (non-suicidal injury)	Na.[i]	Na.	12	Na.

Notes: a) Number of patients randomized corrected for dropout prior to start of treatment; b) Attrition defined as loss due to training cases, drop-out, or not completing the treatment protocol; c) Effect sizes: Cohen's d with pooled SD, within-group calculations, pre-treatment vs. post-treatment or follow-up, bias corrected (Hedges); d) ES stated in paper based on medians; e) Transformed from r to d by the authors; f) SD calculated based on SE and N; g) ES based on completer analysis; N at follow-up (CBT = 10, RST = 11); h) Values for ZAN-BPD not given at follow-up, BEST used instead for ES. i) Measure was not available for measuring ES. * ES (Cohen's d or r) was stated in the paper.

Abbreviations: ST = Schema Therapy; TFP = Transference-Focused Psychotherapy; DBT = Dialectical Behaviour Therapy; SUPT = Supportive Treatment; TAU = Treatment as usual; GPM = General psychiatric management; ECP = Experienced community psychotherapists; MBT = Mentalization-based treatment; SCM = Structured clinical management; DPP = Dynamic deconstructive psychotherapy; MACT = Manual assisted cognitive treatment; STEPPS = System training for emotional predictability and problem solving; BPDSI = Borderline personality disorder severity index, 4th ed.; DIB-R = Diagnostic Interview for BPD-revised; YSQ = Young schema questionnaire; ZAN-BPD = Zanarini rating scale for BPD; BEST = Borderline evaluation of severity over time; BDP-40 = BPD checklist - 40 items.

References:

Bateman, A., & Fonagy, P. (2009a). Randomized controlled trial of outpatient Mentalization-based treatment versus structured clinical management for borderline personality disorder. *American Journal of Psychiatry, 166*, 1355-1364.

Bateman, A., & Fonagy, P. (2009b). Randomized controlled trial of outpatient Mentalization-based treatment versus structured clinical management for borderline personality disorder: Supplementary data. *American Journal of Psychiatry, Retrieved September, 10, 2010, from* http://ajp.psychiatryonline.org/cgi/data/appi.ajp.2009.09040539/DC1/1.

Blum, N., St. John, D., Pfohl, B., Stuart, S., McCormick, B., Allen, J., et al. (2008). Systems training for emotional predictability and problem solving (STEPPS) for outpatients with borderline personality disorder: A randomized controlled trial and 1-year follow-up. *American Journal of Psychiatry, 165*, 468-478.

Bohus M, Dyer AS, Priebe K, Krüger A, Kleindienst N, Schmahl C, et al. (2013) Dialectical behaviour therapy for post-traumatic stress disorder after childhood sexual abuse in patients with and without borderline personality disorder: a randomised controlled trial. *Psychotherapy and Psychosomatics*; 82(4):221–33.

Bos, E. H., van Wel, E. B., Appelo, M. T., & Verbraak, M. J. P. M. (2010). A randomized controlled trial of a dutch version of systems training for emotional predictability and problem solving for borderline personality disorder. *Journal of Nervous and Mental Disease, 198*, 299-304.

Clarkin, J. F., Levy, K. N., Lenzenweger, M. F., & Kernberg, O. F. (2007). Evaluating three treatments for borderline personality disorder: A multiwave study. *American Journal of Psychiatry, 164*, 922-928.

Cottraux, J., Note, I., Yao, S., De Mey-Guillard, C., Bonasse, F., Djamoussian, D., et al. (2008). Randomized controlled comparison of cognitive behavior therapy with Rogerian supportive therapy in chronic post-traumatic stress disorder: A 2-year follow-up. *Psychotherapy and Psychosomatics, 77*, 101-110.

Davidson, K., Norrie, J., Tyrer, P., Gumley, A., Tata, P., Murray, H., et al. (2006). The effectiveness of cognitive behavior therapy for borderline personality disorder: results from the borderline personality disorder study of cognitive therapy (BOSCOT) trial. *Journal of Personality Disorders, 20*, 450-465.

Davidson, K., Tyrer, P., Gumley, A., Tata, P., Norrie, J., Palmer, S., et al. (2006). A randomized controlled trial of cognitive behavior therapy for borderline personality disorder: Rationale for trial, method, and description of sample. *Journal of Personality Disorders, 20*, 431-449.

Farrell, J. M., Shaw, I. A., & Webber, M. A. (2009). A schema-focused approach to group psychotherapy for outpatients with borderline personality disorder: A randomized controlled trial. *Journal of Behavior Therapy and Experimental Psychiatry, 40*, 317-328.

Giesen-Bloo, J., van Dyck, R., Spinhoven, P., van Tilburg, W., Dirksen, C., van Asselt, T., et al. (2006). Outpatient psychotherapy for borderline personality disorder: Randomized trial of Schema-focused Therapy vs. Transference-focused psychotherapy. *Archives of General Psychiatry, 63*, 649–658.

Gregory, R. J., Chlebowski, S., Kang, D., Remen, A. L., Soderberg, M. G., Stepkovitch, J., et al. (2008). A controlled trial of psychodynamic psychotherapy for co-occurring borderline personality disorder and alcohol use disorder. *Psychotherapy, 45*, 28-41.

Gregory, R. J., DeLucia-Deranja, E., & Mogle, J. A. (2010). Dynamic deconstructive psychotherapy versus optimized community care for borderline personality disorder co-occurring with alcohol use disorders: A 30-month follow-up. *Journal of Nervous and Mental Disease, 198*, 292-298.

Linehan, M. M., Comtois, K. A., Murray, A. M., Brown, M. Z., Gallop, R. J., & et al. (2006). Two-year randomized controlled trial and follow-up of dialectical behaviour therapy vs therapy by experts for suicidal behaviors and borderline personality disorder. *Archives of General Psychiatry, 63*, 757-766.

Linehan MM, Korslund KE, Harned MS, Gallop RJ, Lungu A, Neacsiu AD, et al. (2015). Dialectical behavior therapy for high suicide risk in individuals with borderline personality disorder: a randomized clinical trial and component analysis. *JAMA Psychiatry*; 72(5):475–82.

McMain, S. F., Links, P. S., Gnam, W. H., Guimond, T., Cardish, R. J., Korman, L., et al. (2009). A randomized trial of dialectical behavior therapy versus general psychiatric management for borderline personality disorder. *American Journal of Psychiatry, 166*, 1365-1374.

Nadort, M., Arntz, A., Smit, J. H., Giesen-Bloo, J., Eikelenboom, M., Spinhoven, P., et al. (2009). Implementation of outpatient schema therapy for borderline personality disorder with versus without crisis support by the therapist outside office hours: A randomized trial. *Behavior Research and Therapy, 47*, 961-973.

Nordahl, H.M. & Nysæter, T.E. (2005). Schema Therapy for Patients with Borderline personality disorder: A single case series. *Journal of Behaviour Therapy and Experimental Psychiatry*, 36, 254-264.

Priebe S, Bhatti N, Barnicot K, Bremner S, Gaglia A, Katsakou C, et al.(2012). Effectiveness and cost-effectiveness of dialectical behavior therapy for self-harming patients with personality disorder: a pragmatic randomised controlled trial. *Psychotherapy and Psychosomatics;* 81(6):356–65.

Soler, J., Pascual, J. C., Tiana, T., & et al. (2009). Dialectical behaviour therapy skills training compared to standard group therapy in borderline personality disorder: a 3-month randomized controlled clinical trial. *Behaviour Research and Therapy, 47*, 353-358.

Weinberg, I., Gunderson, J. G., Hennen, J., & et al. (2006). Manual assisted cognitive treatment for deliberate self-harm in borderline personality disorder. *Journal of Personality Disorders, 20*, 482-492.
